# Effects of Pulsed Electric Fields (PEF) on Vitamin C and Its Antioxidant Properties

**DOI:** 10.3390/ijms161024159

**Published:** 2015-10-13

**Authors:** Zhi-Hong Zhang, Xin-An Zeng, Charles S. Brennan, Margaret Brennan, Zhong Han, Xia-Yu Xiong

**Affiliations:** 1 College of Light Industry and Food Science, South China University of Technology, Guangzhou 510640, China; E-Mails: zhihong1942@foxmail.com (Z.-H.Z.); fezhonghan@scut.edu.cn (Z.H.); hsiungxiayu@163.com (X.-Y.X.); 2 Centre for Food Research and Innovation, Department of Wine, Food and Molecular Biosciences, Lincoln University, Lincoln 85084, New Zealand; E-Mail: margaret.brennan@lincoln.ac.nz

**Keywords:** vitamin C, PEF, fluorescence, FTIR, HPLC, antioxidant properties

## Abstract

In this study, pulsed electric fields (PEF) treatments and their effects on the structure of vitamin C (VIT-C) were estimated by fluorescence and Fourier transform infrared (FT-IR) spectroscopy, the relative content of VIT-C was measured by HPLC and the antioxidant properties of treated VIT-C by DPPH radical scavenging as well as reducing power tests. The fluorescence intensity of treated VIT-C increased slightly compared to the untreated VIT-C. Moreover, the effect of PEF on the structure of VIT-C was observed using the FT-IR spectra. These phenomena indicated that the PEF affected the conformation of VIT-C, which promoted the VIT-C isomer transformed enol-form into keto-form. In addition, the PEF treatments did not suffer the damage to VIT-C and could slow down the oxidation process in involving of experimental conditions by HPLC. The antioxidant properties of the treated VIT-C were enhanced, which was proved by radical scavenging and also the reducing power tests.

## 1. Introduction

Vitamins are a diverse group of essential organic compounds that participate in normal growth, metabolism and maintenance of life [1,2]. In order to maintain an adequate intake of vitamins, humans must eat a wide variety of food products, either naturally high or, enriched with vitamins or alternatively supplement with multivitamin drugs, because the human body cannot synthesize them or the synthetic amount may not satisfy the bodily needs. Vitamin C (VIT-C) or l-ascorbic acid (AA) is an essential water-soluble vitamin in humans [3,4]. Humans lack the final enzyme (l-gulono-1,4-lactone oxidase) in the VIT-C biosynthesis pathway, thus, humans are dependent on fruits and vegetables (such as orange, sweet potato, and carrot) in their diet to provide VIT-C [5,6]. Vitamin C is beneficial for its antioxidant capacity and stimulation of immune system [7,8,9].

Although most vegetables and fruits contain certain amount of VIT-C, various storage conditions and processing methods can significantly affect VIT-C content [10], because VIT-C is a thermally sensitive bioactive nutrient when in the presence of oxygen [11,12,13,14,15,16]. Some advanced non-thermal methods, therefore are used in fruit and vegetable processing in order to maintain nutrition, such as ultra-high pressure processing (UHP) [17,18], pulsed electric fields (PEF) [19,20,21].

PEF technology involves the application of very short pulses (1–10 μs) of high electric intensity (10–100 kV/cm) to treat food products placed between two electrodes without substantially heating the product [22,23]. Nowadays, it is one of the hot points regarding the effect of PEF technology on nutrient, especially, vitamins and polyphenols [11,12,13,24]. Ade-Omowaye *et al.* [25] showed that the VIT-C retention of red bell pepper immediately after PEF treatment (50 pulses at 2 kV/cm, pulse duration 400 ms) ranged from 89.6% to 96.5%. Odriozola-Serrano *et al.* [20] observed that the VIT-C retention of strawberry juice was 98% after High Intensity Pulse Electric Field (HIPEF) (35 kV/cm, 1000 μs, and monopole mode). Moreover, they found that VIT-C retention was related to some parameters in terms of frequency, electric field intensity, pulsed width and polarity mode by a response surface methodology. In addition, many studies have proven that the retention of VIT-C was higher after PEF treatments than after thermal treatments [20,21,26]. In previous studies, it has been reported that the PEF-processing resulted in increased VIT-C retention when compared to other thermal treatments [20,21,27].

As mentioned above, there are some ambiguous results in effect of PEF treatment on VIT-C properties in previous studies. In addition, the complex food structures of fabricated foods have made it difficult to determine the effect of PEF treatment on the VIT-C. Relatively few studies mentioned that the PEF treatment directly affected the VIT-C. To solve the problem, systematically characterizing the effect of PEF treatment involved in different electric field strength and treatment time on the structure of VIT-C by fluorescence and Fourier transform infrared (FT-IR) spectroscopy is attempted. Moreover, the effect of PEF treatment on relative content of VIT-C was determined by high performance liquid chromatography (HPLC) and the antioxidant properties were measured by DPPH radical scavenging and the reducing power test.

## 2. Results and Discussion

### 2.1. Fluorescence of VIT-C

**Figure 1:**
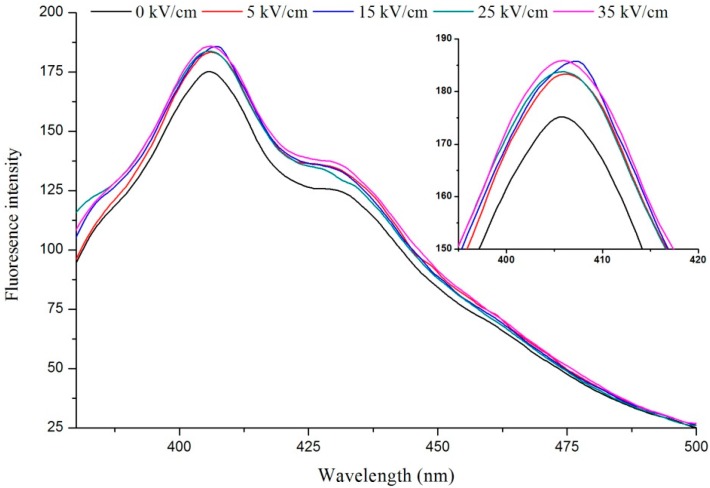
The effect of different electric field strength at 9 min on fluorescence of vitamin C (VIT-C).

**Figure 2:**
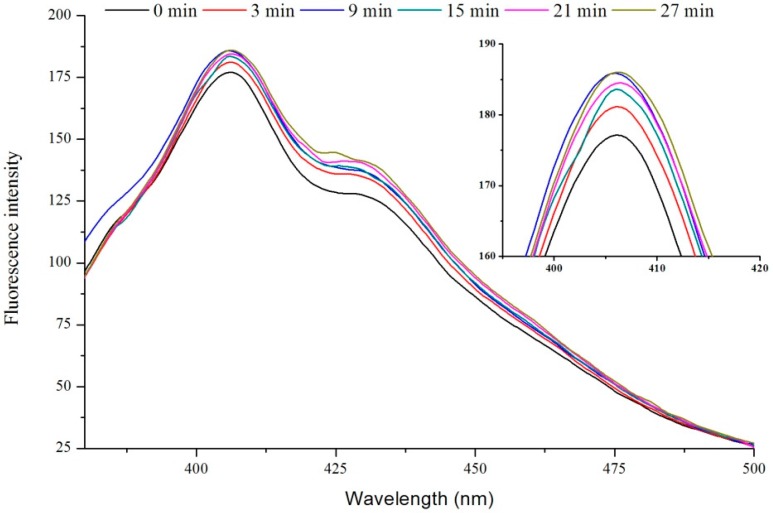
The effect of different pulsed electric fields (PEF) treatment time at 35 kV/cm electric field strength on fluorescence of VIT-C.

The effects of different electric field strength and treatment time on fluorescence of VIT-C are shown in Figure 1 and Figure 2, respectively. Previously researchers have illustrated that the condensation product emits strong fluorescence and that the detection limit is very low (0.006 μg/mL) [28]. Therefore, fluorescence of VIT-C was determined based on the condensation reaction between VIT-C and *o*-phenylenediamine. This method can not only determine the content of VIT-C, but also observe the effects of PEF on the structure of VIT-C. It was observed that the fluorescence intensity of PEF treated VIT-C was higher than untreated VIT-C at 405 nm, and showed a tendency that the higher electric field strength was corresponding to the stronger fluorescence intensity. Moreover, the PEF treatment time also affected the fluorescence intensity of VIT-C. The fluorescence intensity of VIT-C treated by 3, 9, 15, 21 and 27 min (the effective PEF treatment was 0.8, 2.4, 4.0, 5.6, and 7.2 ms) were higher than the untreated (0 ms) sample at 405 nm, and the fluorescence intensity gradually increased with the treatment time. This phenomenon indicated that the PEF treatment could exert influence on the condensation reaction process of VIT-C and *o*-phenylenediamine, which is due to the type of VIT-C isomer present.

**Figure 3:**
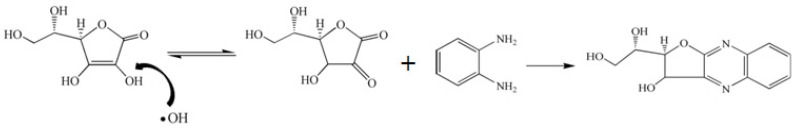
The possible mechanism of condensation reactions of VIT-C with *o*-phenylenediamine by PEF treatments.

Wu *et al*. (2003) [28] reported that hydroxyl ion (–OH) could promote the conversion of VIT-C isomer from enol-form to keto-form in the range of pH 9.3–9.5, which proved that alkaline condition was conducive to the condensation reaction. In this study, the reaction system was under acidic conditions (pH < 7.0), but the fluorescence intensity was increased by PEF treatment, which indicated that PEF treatment promoted the conversion of VIT-C isomer. It has been reported that the water molecule was easily polarized by PEF and was dissociated into ions at high field strength [29]. The presence of polar groups can react with the interior macromolecules or active ingredients. Zhang *et al.* [30] reported that the PEF processing generated hydrogen radicals in phosphate buffer and oleic acid emulsion, they also illustrated that the electric field strength had a significant effect on H_2_O_2_ concentrations (*p* < 0.5) when estimated using electron spin resonance (ESR) techniques. Thus, it was believed that the free radicals generated by PEF treatment attacked the hydroxyl, which belonged to the VIT-C second carbon atom to complete the conversion of configuration. The possible mechanism is illustrated in Figure 3. This theory was consistent with the experimental phenomenon of VIT-C degradation by PEF processing in previous studies [21,31]. Moreover, some previous studies reported that the PEF treatment could influence the structure of amino acids, proteins and polysaccharides, which mainly related to the weak bonds such as hydrogen bonds, disulfide bonds and hydrophobic bonds [32,33,34].

### 2.2. FT-IR Analysis

**Figure 4:**
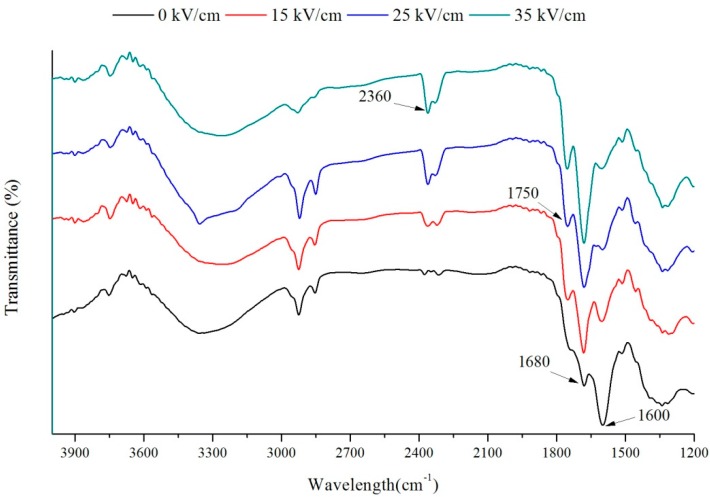
Fourier transform infrared (FTIR) spectra of VIT-C solution by PEF treatments.

The FT-IR spectra of PEF treated VIT-C solution are shown in Figure 4. The infrared spectroscopy characteristic absorption band of the VIT-C mainly appeared in wavenumber as follows. The band at 1600 cm^−1^ is contributes to vibration of C=C bonds. The band at 1680 cm^−1^ causes vibration of the C=O area present in the lactone ring system. Data presented in this manuscript illustrates that the stretching vibration of C=C and C=O, respectively, appeared at about 1700 and 1660 cm^−1^ by FT-IR spectra recorded in KBr pellets, this is consistent with previous studies when the effect of the solvent is taken into consideration [35,36].

The characteristic absorption band of the VIT-C showed some changes due to PEF treatment from the FT-IR spectra. When the electric field strength increased, the intensity of the band at 1600 cm^−1^ decreased, while the intensity of the band at 1680 cm^−1^ increased compared with untreated VIT-C. This phenomenon indicated that the structure of VIT-C has been transformed from the enol-form to keto-form by PEF treatment. Moreover, an obvious absorption peak was located at the band of 2360 cm^−1^, which was attributed to the O–H stretching vibration. It indicates that the hydrogen atom from the O–H on third carbon was generated with carbonyl on second carbon atom bonded to a five membered ring system. Thus, it is believed that the PEF treatment could influence the structure of VIT-C from the FT-IR spectra. In addition, other previous studies also identified that the PEF could affect the structures of the protein and chitosan [37,38].

### 2.3. The Relative Content of VIT-C

When the PEF treatment affects the VIT-C content it is reflected through a change in the peak area of VIT-C by HPLC. The effect of PEF treatment on the relative content of VIT-C measured by HPLC is shown in Table 1. The relative content of PEF treated VIT-C was 97.56, 97.98, 93.60 and 95.28 mg/L at 5, 15, 25 and 35 kV/cm for 3 min, which was apparently higher than the untreated VIT-C of 93.96 mg/L. The results also showed that the VIT-C content of PEF treated samples was higher than the untreated VIT-C samples, also the untreated samples decreased in VIT-C over the increasing treatment time. This result was in agreement with the findings of Odriozola-Serrano *et al.* [21], who reported that the PEF treatment exhibited higher VIT-C retention than heat treatments in different fruits and vegetables juice. Moreover, some other studies showed that the 35 kV/cm of PEF treatment exhibited higher effectiveness on the retention of VIT-C than other selected electric field strengths [20] or thermal treatments [39,40]. The results showed that the content of VIT-C decreased with the increasing treatment time under 5 and 15 kV/cm, which was in agreement with the finding of Elez-Martinez and Martin-Belloso [41]. Meanwhile, a very interesting phenomenon was noted at 35 kV/cm and that was that the relative content of VIT-C increased from 95.28 to 98.14 mg/L as time increased from 3 to 27 min. A previous study reported that the ascorbic acid of orange-carrot juice decreased from 25.07 ± 0.68 to 22.53 ± 1.192 mg/100 mL with an increase in treatment time from 90 to 200 μs at 35 kV/cm [31]. The reason for this phenomenon may be due to the difference of the reaction conditions such as pH, electrical conductivity and temperature. These results indicate that whether using PEF treatment or not, the content of VIT-C could reduce as a consequence of extending the processing time which can be due to the natural oxidation of VIT-C. Moreover, the PEF treatments did not cause the damage to VIT-C and were able to retard the oxidation process under the experimental conditions created.

**Table 1:** The effect of PEF treatment on the relative content of VIT-C as detected by HPLC.

Relative Content (mg/L)	0 kV/cm	5 kV/cm	15 kV/cm	25 kV/cm	35 kV/cm
3 min	93.69 ± 0.12 ^A a^	97.56 ±0.10 ^B C a^	97.98 ± 0.38 ^C a^	93.60 ± 0.81 ^A a^	95.28 ± 0.24 ^D a^
9 min	92.85 ± 0.23 ^A b^	98.12 ± 0.31 ^B a b^	97.96 ± 0.50 ^B a^	96.77 ± 0.24 ^C b^	96.30 ± 0.56 ^C a^
15 min	91.72 ± 0.41 ^A c^	98.53 ± 0.27 ^B b^	97.77 ± 0.43 ^B a^	98.45 ± 0.62 ^B c^	96.50 ± 0.70 ^C a^
21 min	91.51 ± 0.15 ^A c^	97.43 ± 0.42 ^B a^	97.59 ± 0.41 ^B a^	98.33 ± 0.47 ^B c^	98.19 ± 0.53 ^B b^
27 min	91.38 ± 0.22 ^A c^	95.12 ± 0.51 ^B c^	97.32 ± 0.35 ^C a^	98.01 ± 0.53 ^C b c^	98.14 ± 0.42 ^C b^

PEF: pulsed electric fields; VIT-C: vitamin C. Values with different letters in the column (a–c) and in the row (A–D) mean significant difference (*p* < 0.05).

### 2.4. 1,1-Diphenyl-2-picrylhydrazyl (DDPH) Radical Scavenging Activity

**Figure 5:**
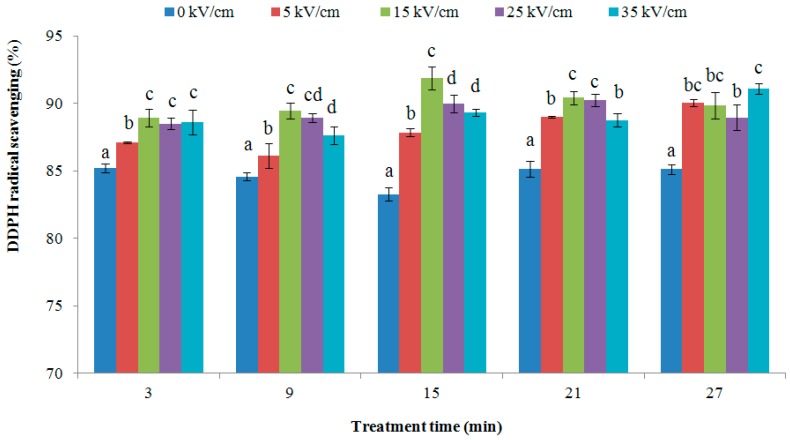
1,1-diphenyl-2-picrylhydrazyl (DDPH) radical scavenging activity of VIT-C by PEF treatments. Values in columns followed by different letters mean significant difference (*p* < 0.05).

Figure 5 illustrates that the 1,1-diphenyl-2-picrylhydrazyl (DDPH) radical scavenging ability of PEF treated VIT-C increased compared to untreated VIT-C. The DDPH radical scavenging of VIT-C was 87.84%, 91.89%, 89.99% and 89.32%, respectively, with 5, 15, 25 and 35 kV/cm of PEF treatment for 15 min; these were all a significant increase compared with untreated VIT-C, which had a DDPH radical scavenging activity of 83.28% (*p* < 0.05). However, the DPPH radical scavenging activity that was treated for 3 min with different electric field strengths was not significantly different at 15–35 kV/cm. This result indicated that the treatment time was obvious influence on the DPPH radical scavenging activity. As treatment time increased, the DPPH radical scavenging activity of non-treated VIT-C was obviously decreased, but the PEF treatment of VIT-C caused DPPH radical scavenging activity to show a trend of either no reduction or some increase. These results illustrated that the PEF treatment at appropriate electric field strength and treatment time could effectively maintain the activity of VIT-C. Similar results were found in a previous study by Wang and coworkers [38]. In this context, the antioxidant activity of glutathione (GSH) treated with different electric field intensities from 10 to 30 kV/cm were significantly increased, especially at the electric field strength of 10 kV/cm. Other previous studies have also indicated that under certain electric field strengths, the structure of protein showed some changes, such as the molecular weight, the quaternary structure, and polarization of the proteins molecule, which caused the increase or decrease of the DDPH radical inhibition [42,43]. It can be shown that the PEF treatment influenced the structure of VIT-C in Section 3.1 and Section 3.2, which may be one of the reasons for the increasing DPPH radical scavenging activity. In addition, the PEF treated solutions could exhibit the higher VIT-C retention (95.12–98.53 mg/L), which may be one of the reasons for enhancing the DPPH radical scavenging.

### 2.5. Reducing Power Ability

**Figure 6:**
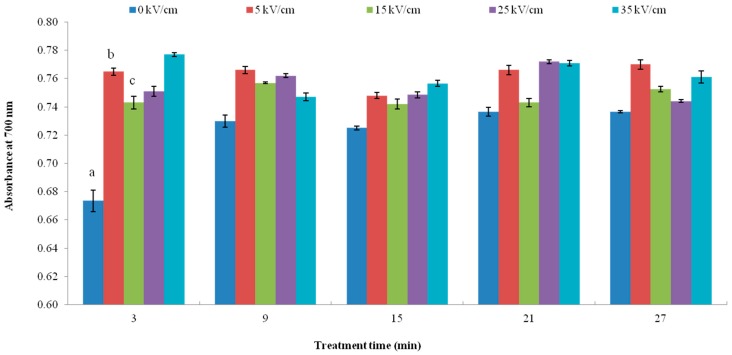
Reduce power activity of VIT-C by PEF treatments. Values in columns followed by different letters mean significant difference (*p* < 0.05).

The reducing power activity of PEF treated VIT-C using the potassium ferricyanide reduction method is shown in Figure 6. The results were expressed as absorbance at 700 nm. It is shown that the reducing power ability, by different electric field strengths based on 3 min treatment of PEF treated VIT-C, significantly increased as compared with non-treated VIT-C (*p* < 0.05), reducing power increased by 13.50%, 10.24%, 11.42%, and 15.28% at 5, 15, 25 and 35 kV/cm, respectively. Among the different electric field strengths, however, there was no significant difference. At other processing times, the PEF treated VIT-C also showed a tendency for the reducing power ability to increase as compared to non-treated VIT-C. These results indicate that the reducing power of PEF treated VIT-C can be increased compared with non-treatment. The reason for this phenomenon was that the PEF treatment altered the structure and polarization of VIT-C. As a result, it is easier for the VIT-C to provide electrons, thereby the antioxidant capacity was enhanced; similar results were found in the previous study [44]. In this study, the treatment time was extended and the reducing power of peptides derived from egg white was increased by treatment with 10 kV/cm PEF. Notably, the reducing power was increased by 0.118 after a 5 h PEF treatment compared to an untreated sample of 0.646. This result showed that the PEF treatment time could significantly increase the antioxidant activity of the peptides. It was believed that PEF treatment could activate the antioxidant activity of peptides derived from egg white by polarization of peptide molecules, and destroy the non-covalent bonds, by hydrophobic interactions, electrostatic interactions and creating hydrogen bonds [44]. In addition, a previous study observed the effects of different electrode materials on antioxidant capacity and other characteristics of cyaniding-3-glucoside (Cy-3-glc) and cyaniding-3-sophoroside (Cy-3-sop) during 10 kV/cm of PEF treatment. The results showed that the antioxidant of Cy-3-glc and Cy-3-sop was increased, regardless of the method used (FRAP, DDPH and ORAC), which was due to the higher retention of Cy-3-sop and Cy-3-glc, the electrochemical reaction and forming of anthocyanin–metal complexes that have higher antioxidant capacity [45].

## 3. Materials and Methods

### 3.1. Materials

Vitamin C and ethanol were purchased from Sinopharm Chemical Reagent Co., Ltd. (Shanghai, China). Potassium ferricyanide purchased from Tianjin Fuchen Chemical Reagent Factory (Tianjin, China). *O*-phenylenediamine was obtained from Tianjin Kemiou Chemical Reagent Co., Ltd. (Tianjin, China). Methanol was obtained from ANPEL Laboratory Technologies Inc. (Shanghai, China). 1,1-Diphenyl-2-picrylhydrazyl (DDPH radical) was obtained from Sigma-Aldrich, Chemie GmbH (St. Louis, MO, USA). All other reagents and chemicals used were of analytical grade. All reagents were prepared with deionized water (conductivity 0.3 μS/cm).

### 3.2. PEF Treatment System

**Figure 7:**
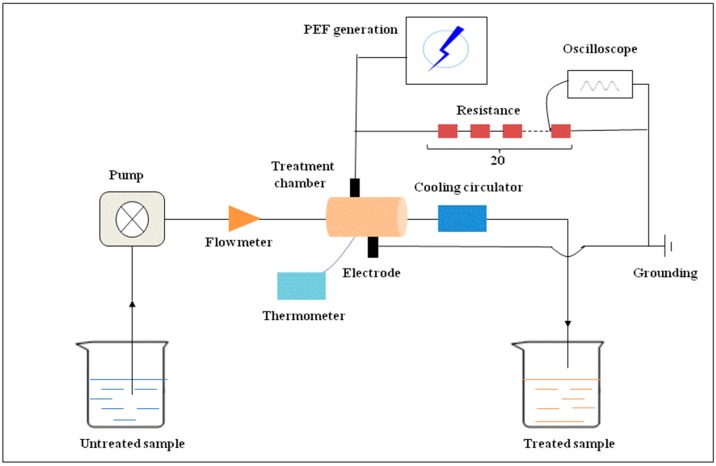
The scheme of the PEF treatment system used in the experiment.

The continuous PEF system was designed and manufactured by South China University of Technology PEF team (SY-Z-500; Guangzhou, China). The structure of the equipment has been reported in previous studies [46,47]. The main parameters of this equipment were as follows: frequency of 1 kHz, pulse width of 40 μs, unipolar square-wave. A two channel digital storage oscilloscope (DST1102B, Tekway Technologies Co., Ltd., Nanjing, China) was used to monitor the power supply of PEF system during the treatment. The treatment chamber was made of Teflon. The parameters of chamber were as follows: the volume of 0.02 mL, the distance between two parallel titanium-based alloy electrodes of 0.3 cm. The sample flow was controlled by a peristaltic pump (323 E/D, Watson Marlow, NC, USA). The sample temperature was measured by thermocouple (EW-981, Ewelly, Guangzhou, China) and was maintained by a cooling circulator (DLSK 3/10, Ketai Laboratory Equipment Co., Ltd., Zhengzhou, China). The scheme of the PEF treatment system used in the experiment is shown in Figure 7. In this study, 100 mg/L of VIT-C solution was prepared by ethanol-aqueous (volume ratio of 1:1) in a 1 L volumetric flask. The bubbles were removed by vacuum pump before the treatment. The pH of VIT-C solution was 4.47 and conductivity value was 10.0 mS/cm (20 ± 1 °C). The flow rate of sample was set at 60 mL/min by a peristaltic pump and the temperature was maintained at 20 ± 1 °C by cooling circulator. According to Equations (1) and (2), the effective treatment time (*t*) of PEF were calculated to be 0.8, 2.4, 4.0, 5.6 and 7.2 ms inthe different cycle times (1, 3, 5, 7, and 9), corresponding to the sample actual treatment time was 3, 9, 15, 21 and 27 min, respectively. In addition, the selected electric field strengths were 0, 0.5, 12.5, 25 and 35 kV/cm. The energy input (*Q*) was calculated by Equation (3) as described by Han *et al.* [32], and the results are shown in Table 2. After PEF treatment, each sample was collected in a 10 mL glass test tube with stopper and stored at 4 ± 1 °C until analysis could be carried out. The experiments were performed three times.

(1) $$t=N_{t}\times W_{p}\times n$$

(2) $$N_{t}=\frac{f\times V}{S}$$

where *N_t_* is pulse number, *W_p_* represents pulse width (μs), *n* denotes sample cycles, *f* represents pulse repetition rates, *V* is the volume of the chamber (mL) and *S* is the flow rate (mL/s).

(3) $$Q=E^{2}\times t\times \text{δ}$$

where *Q* is the energy input (J/m^3^), *E* is the electric field strength (V/m), *t* is the treatment time and δ is the conductivity of the sample (S/m).

**Table 2:** The energy input (*Q*, kJ/cm^3^) of PEF in the different treatment condition.

Electric Field Strength (kV/cm)	Treatment Time (ms)				
	0.8	2.4	4.0	5.6	7.2
5	0.05	0.15	0.25	0.35	0.45
15	0.45	1.35	2.25	3.15	4.05
25	1.25	3.75	6.25	8.75	11.25
35	2.45	7.35	12.25	17.15	22.05

### 3.3. Fluorescence Measurement

The determination of VIT-C was performed as described by Wu *et al.* [28] with some modifications. The VIT-C content was determined by a spectrofluorometer (Perkin–Elmer LS55, Beaconsfield, Bucks, UK). Briefly, 0.5 mL of sample was added to 4.5 mL of ethanol-aqueous (volume ratio of 1:1), and then mixed with 0.5 mL *o*-phenylenediamine solution (0.2 g/L). The mixture was placed in dark and allowed to stand for 30 min. Fluorescence intensities of samples were determined in a 1 cm quartz cell (excitation and emission wavelengths of 355 and 405 nm, respectively). Width of excitation and emission slit were both set at 10 nm, and the relative fluorescence was determined.

### 3.4. FT-IR Analysis

Spectrometry analysis of the VIT-C solutions by PEF treatment was carried out to observe the structure by using an FT-IR spectrometer (Vector33, Bruker, Ettlingen, Germany). The samples were fixed in the CaF_2_ window and FTIR spectra were collected in the range of 1100–4000 cm^−1^.

### 3.5. Determination of VIT-C by HPLC 

The relative content of VIT-C was determined using Waters 1525 Binary HPLC (Waters Corp., Milford, MA, USA) equipped with a C_18_ column (250 × 4.0 mm, 5 μm, SunFire, Waters, Milford, MA, USA), an autosampler (2707, Waters, Milford, MA, USA) and a photodiode array detector (2998, Waters, Singapore). The method described by Klimczak and Gliszczyńska-Świgło [48] with some modifications. A gradient of mobile phase composed of water (solvent A) and methanol (solvent B) was used 10% A in 15 min with the flow rate of 0.8 mL/min. The eluate was detected by UV detection at 245 nm. The injection volume was 20 μL.

### 3.6. DDPH Radical Scavenging Activity

The antioxidant activity of VIT-C solutions was determined by a stable capacity DDPH radical following the method described by *Gu* *et al.* [49] with some modifications. Sample (200 mL) was mixed with 3.5 mL of 0.1 mmol/L DPPH solution (DPPH was dissolved in ethanol) and vortexed for mixing before leaving the samples to stand for 20 min in the dark. Absorbance values were determined at 517 nm using a spectrophotometer (UV-1800, SHIMADZU, Tokyo, Japan). DPPH activity was calculated by the following Equation (4).

(4) $$\text{SA}(\%)=(1-\frac{{\text{A}}_{1}-{\text{A}}_{2}}{{\text{A}}_{3}})\times 100$$

where SA is the scavenging activity DDPH radical. A_1_ is the absorbance of the sample. A_2_ is the absorbance of the blank. A_3_ is the absorbance of the control.

### 3.7. Reducing Power Ability

The reducing power of sample was determined by the method of Yen *et al.* [50] with some modifications. The sample solutions were mixed with 2.5 mL of 0.2 mol/L phosphate buffer (pH 6.6) and 2.5 mL of 1% potassium ferricyanide. Each sample solution was incubated for 20 min at 50 °C and then cooled to room temperature. Trichloroacetic acid (2.5 mL) was added to each sample, followed by vigorous mixing. An aliquot of 2.5 mL of each sample was mixed 0.5 mL of 1 g/L ferric chloride and the absorbance of the resultant solution was measured at 700 nm. Higher absorbance of the reaction mixture indicated greater reducing power.

### 3.8. Statistical Analysis

All results were reported as mean ± standard deviation (*n* = 3) using SPSS 16.0 software (IBM, New York, NY, USA). Variance analysis and graphs were obtained by Origin 8.0 software (OriginLab, Massachusetts, MA, USA) to determine the different treatment variations. Significance testing was performed by Tukey’s multiple-comparison test and the differences were statistically significant. The significance level was 5% (*p* < 0.05) unless otherwise stated.

## 4. Conclusions

The effects of PEF treatment on VIT-C structure, and its relative content and antioxidant properties were investigated in this study. The fluorescence intensity increased by PEF treatment as compared to the non-treated VIT-C using the fluorescence measurement. This result indicated that the PEF treatment could modify the molecular conformation of VIT-C (from enol-form to keto-form). FT-IR spectra proved the changes of VIT-C structure by PEF treatment. Moreover, the PEF treatments did not damage the VIT-C and could slow down the oxidation process in involving of experimental conditions. In addition, PEF treatment could enhance the antioxidant properties through the DPPH radical scavenging and reducing power test, which could be due to the structure changes of VIT-C. The antioxidant properties showed that the DPPH radical scavenging was significantly increased by 4.56%, 8.61%, 6.71%, and 6.04%, respectively, which were corresponding to 5, 15, 25, and 35 kV/cm under 3 min processing time compared to the non-treated VIT-C (*p* < 0.05).
